# Adapting to ever-changing conditions

**DOI:** 10.7554/eLife.91717

**Published:** 2024-02-28

**Authors:** Serge Pelet

**Affiliations:** 1 Department of Fundamental Microbiology, University of Lausanne Lausanne Switzerland

**Keywords:** stress adaptation, glucose starvation, systems biology, microfluidics, *S. cerevisiae*

## Abstract

Experiments involving periodic stimuli shed light on the interplay between hyper-osmotic stress and glucose starvation in yeast cells.

**Related research article** Duveau F, Cordier C, Chiron L, LeBec M, Pouzet S, Séguin J, Llamosi A, Sorre B, Di Meglio JM, Hersen P. 2024. Yeast cell responses and survival during periodic osmotic stress are controlled by glucose availability. *eLife* **12**:RP88750. doi: 10.7554/eLife.88750.

Microbial cells have to constantly adapt to their environment. Consider, for example, yeast cells growing on the surface of a fruit: if the fruit is suddenly exposed to direct sunlight, the yeast cells will have to cope with a rise in temperature, DNA damage (caused by the ultraviolet radiation in the sunlight), and an increase in the osmolarity of their growth medium (caused by accelerated evaporation of the medium). To adapt to such changes, microbial cells have evolved complex signal transduction pathways, which allow them to adjust their metabolism and produce stress-response proteins that help the cells adapt to their new environment and pursue their growth. While the key molecular players involved in these processes have been identified, many of the details are not fully understood, especially when the cells have to respond to two or more changes in their environment.

In the laboratory, the budding yeast *Saccharomyces cerevisiae* has been used as a model system to understand the response of eukaryotic cells to changes in the environment (Ho and Gasch, 2015). In most of these experiments, the researchers modified one environmental parameter at a specific time, which made it possible to identify the diverse components that relay information from the cells’ environment into a defined biological response. These experiments revealed that the different signal transduction pathways involved in the various responses are connected to each other and form a complex network. However, to better understand this network, it is necessary to perform experiments in which multiple stimuli are combined in a dynamic way. Microfluidic devices consisting of micrometer-sized growth chambers fed by flow channels are ideally suited for such experiments (Breslauer et al., 2006; Hersen et al., 2008). Now, in eLife, Pascal Hersen and colleagues – including Fabien Duveau as first author – report how they used microfluidic chips to monitor the response of yeast cells to repeated periods of hyper-osmotic stress and/or glucose starvation for up to 24 hours (Duveau et al., 2024).

Hyper-osmotic stress activates a kinase called Hog1, which orchestrates cellular adaptation, notably by stimulating the production of glycerol (Brewster and Gustin, 2014). During the 30 minutes after the onset of hyper-osmotic stress, the yeast cells accumulate glycerol until the internal osmotic pressure reaches a new equilibrium with the external osmotic pressure and growth can resume. In a fluctuating environment, when hyper-osmotic stress is applied repeatedly to cells for periods of less than 30 minutes, the cells cannot reach the equilibrium and growth is inhibited during the exposure to the stress. In contrast, if the frequency of the fluctuations is reduced and the stress is applied for longer than 30 minutes, the cells will be able to start growing again once equilibrium is re-established (Figure 1A). Thus, fast (high frequency) fluctuations in hyper-osmotic stress have a greater impact on growth than slow (low frequency) fluctuations.

**Figure 1.**
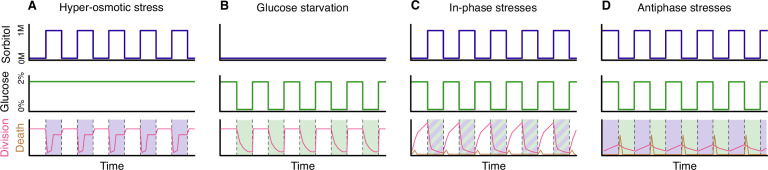
Yeast cells under stress. (**A**) Yeast cells subjected to periodic hyper-osmotic stress (top panel) and provided with a steady supply of glucose (middle panel) display high rates of cell division (red line, bottom panel) in the absence of stress. During periods of stress (shaded area, bottom panel), which last 48 minutes in this example, the rate of cell division drops sharply at first, but then begins to increase after about 30 minutes as the accumulation of glycerol brings the internal and external osmotic pressures back into equilibrium. The rate of cell division then plateaus, before increasing again to its normal value once the hyper-osmotic stress is removed. Hyper-osmotic stress is applied by exposing yeast cell to a medium containing an elevated concentration of a sugar called sorbitol. (**B**) Yeast cells subjected to periodic glucose starvation (middle panel) suffer a progressive arrest of cell division (red line, bottom panel). However, cell division returns quickly to normal rates once glucose becomes available again. (**C**) When yeast cells are subjected to periodic hyper-osmotic stress that is “in-phase” with periodic glucose starvation, the rate of cell division drops sharply during the period when both stresses are being applied, but it recovers when these stresses are removed (red line, bottom panel). However, a small number of cells deaths occur when the stresses are removed (brown line, bottom panel). (**D**) When the two stresses are applied at different times (the antiphase experiments), the overall rate of cell division is low (red line, bottom panel), and the number of cell deaths due to lysis is higher (brown line, bottom panel) than in the in-phase experiments.

The impact of glucose starvation is different. When glucose is removed, the rate of growth falls rapidly but cell division can still take place during the 20–30 minutes before the cell cycle is blocked (Broach, 2012; Irvali et al., 2023). This means that repeatedly starving the cells of glucose for short periods of time has a relatively low impact on growth, whereas starving them for periods of longer than 30 minutes will bring growth to a halt until glucose is replenished. Therefore, in contrast to what is seen with hyper-osmotic stress, slow fluctuations in glucose starvation have more impact on growth than fast fluctuations.

In follow-up experiments, Duveau et al. – who are based at the Institut Curie and other institutes in Paris and Lyon – tested a combination of the two stresses being applied periodically. First, hyper-osmotic stress and glucose starvation were applied at the same time. In these “in-phase” experiments, only a few cell divisions were observed when the stresses were being applied, but growth restarted when the stresses were removed (Figure 1C). However, removal of the stresses also resulted in rare events of cell death.

Next, periods of hyper-osmotic stress without glucose starvation were followed by periods of glucose starvation without hyper-osmotic stress. In these “antiphase” experiments, the overall number of cell divisions was lower than in the in-phase experiments, and the rate of cell death was higher (Figure 1D). The surprisingly low number of cell divisions during the periods of hyper-osmotic stress – when glucose was present – was likely caused by the high osmolarity in the cells disrupting the process of glycolysis. Cells can only produce the glycerol needed for stress adaptation if glycolysis is active (Hohmann et al., 2007), but high levels of osmolarity can disrupt many cellular functions, including glycolysis (Miermont et al., 2013). Cells are thus trapped between two conflicting imperatives, and it would be interesting to explore if a small delay between the replenishment of glucose and the application of the hyper-osmotic stress could provide more favourable conditions for cellular adaptation and thus lead to increased growth.

Another key observation is that the substantial cell death via lysis that is observed in the anti-phase experiments happens mostly at the start of the period of glucose starvation (Figure 1D). Remarkably, yeast cells that cannot accumulate glycerol during period of hyper-osmotic stress (for instance, mutants deleted for the kinase Hog1) do not undergo lysis. Therefore, under the specific conditions tested in these experiments, a mutant that would not survive in the wild becomes fitter than wild-type cells which have evolved to withstand a wide range of stressful situations.

The interplay between hyper-osmotic stress and glucose starvation is intricate because the kinase Hog1, when active, diverts part of the cellular glucose pool towards the production of glycerol. Interestingly, in a low-glucose medium, the stress adaptation takes much longer, which suggests that a smaller quantity of glucose is devoted to glycerol production (Shen et al., 2023). The generation of more complex temporal stress stimuli could help identify the parameters that control the metabolic fluxes between growth and stress adaptation. It would also be valuable to include other kinds of stresses in such experiments. In particular, studying the interplay between hyper-osmotic stress and nitrogen starvation could help us understand which of the processes seen by Duveau et al. are specific to glucose metabolism and which are relevant to nutrient starvation more generally.
